# Fan Cells in Layer 2 of the Lateral Entorhinal Cortex Are Critical for Episodic-like Memory

**DOI:** 10.1016/j.cub.2019.11.027

**Published:** 2020-01-06

**Authors:** Brianna Vandrey, Derek L.F. Garden, Veronika Ambrozova, Christina McClure, Matthew F. Nolan, James A. Ainge

**Affiliations:** 1 Centre for Discovery Brain Sciences, University of Edinburgh, Hugh Robson Building, 15 George Square, Edinburgh EH8 9XE, Scotland; 2 School of Psychology & Neuroscience, University of St. Andrews, St. Mary’s Quad, South Street, St. Andrews KY16 9JP, Scotland

**Keywords:** recognition memory, associative memory, hippocampus, Alzheimer’s, medial entorhinal cortex, object recognition, fan cells, lateral entorhinal cortex, episodic memory

## Abstract

Episodic memory requires different types of information to be bound together to generate representations of experiences. The lateral entorhinal cortex (LEC) and hippocampus are required for episodic-like memory in rodents [1, 2]. The LEC is critical for integrating spatial and contextual information about objects [2, 3, 4, 5, 6]. Further, LEC neurons encode objects in the environment and the locations where objects were previously experienced and generate representations of time during the encoding and retrieval of episodes [7, 8, 9, 10, 11, 12]. However, it remains unclear how specific populations of cells within the LEC contribute to the integration of episodic memory components. Layer 2 (L2) of LEC manifests early pathology in Alzheimer’s disease (AD) and related animal models [13, 14, 15, 16]. Projections to the hippocampus from L2 of LEC arise from fan cells in a superficial sub-layer (L2a) that are immunoreactive for reelin and project to the dentate gyrus [17, 18]. Here, we establish an approach for selectively targeting fan cells using Sim1:Cre mice. Whereas complete lesions of the LEC were previously found to abolish associative recognition memory [2, 3], we report that, after selective suppression of synaptic output from fan cells, mice can discriminate novel object-context configurations but are impaired in recognition of novel object-place-context associations. Our results suggest that memory functions are segregated between distinct LEC networks.

## Highlights

• Sim1:Cre mice provide access to DG-projecting fan cells in lateral entorhinal cortex
• Fan cells are not required for novel object or object-context recognition
• Fan cells are required to discriminate novel object-place-context configurations
• Episodic-like memory impairment is correlated with extent of fan-cell inactivation

## Teaser

Vandrey et al. show that synaptic inputs into the dentate gyrus from fan cells in layer 2 of the lateral entorhinal cortex are required for episodic-like memory in mice, providing evidence of dissociable memory functions in LEC networks.

## Results

### Sim1:Cre Mice Give Genetic Access to Fan Cells in L2 of LEC

**Figure 1:**
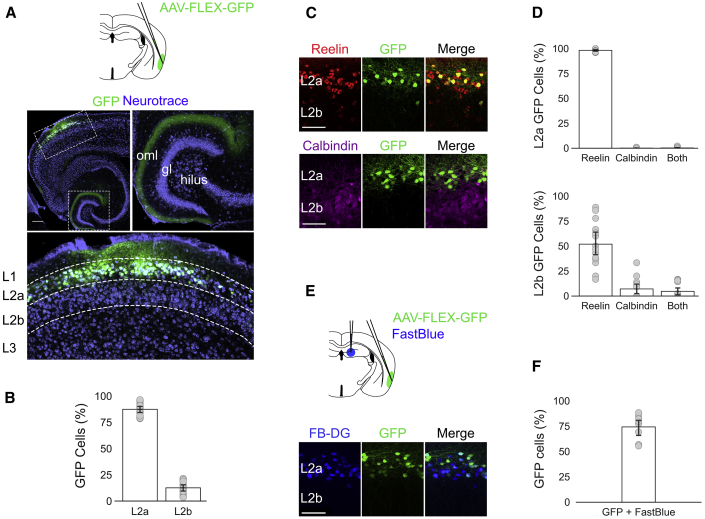
Sim1:Cre Mice Provide Genetic Access to Reelin Cells in L2 of LEC that Project to the Dentate Gyrus (A) Schematic for targeting superficial LEC with AAV-FLEX-GFP and images of a horizontal brain section from a Sim1:Cre mouse showing GFP expression (green) counterstained with Neurotrace (violet). The dashed boxes in the top left image indicate regions shown in the insets. Axonal labeling is found in the outer molecular layer of the dentate gyrus (top right) and L2 of LEC (bottom). gl, granule cell layer; oml, outer molecular layer. The scale bar represents 250 μm. (B) Percentage of neurons that expressed the reporter gene that were in L2a (left, n = 4 mice; 87.4% ± 1.6%; 1,282/1,490 cells; 14 sections) and L2b (right, 12.6% ± 1.6%; 207/1,490 cells) of the LEC. Grey dots indicate percentage values calculated for each section of tissue. Error bars represent SEM. (C) Examples of AAV-FLEX-GFP-labeled cells that are positive for reelin (red, L2a: 98.3% ± 0.4%, 1,260/1,282 cells; L2b: 52.0% ± 5.7%, 97/207 cells), but not calbindin (purple, L2a: 0.1% ± 0.1%, 1/1,282 cells; L2b: 7.1% ± 2.8%, 17/207 cells). Scale bars represent 100 μm. See also Figures S1 and S2. (D) Proportion of neurons expressing the reporter gene (GFP) that were labeled by staining with antibodies against reelin and calbindin. Grey dots indicate percentage values calculated for each section of tissue. Error bars represent SEM. (E) Cells labeled by injection of retrograde tracer (blue) overlap with cells labeled by injections of AAV-FLEX-GFP. Scale bar represents 100 μm. Schematic shows the injection strategy used to target superficial LEC with AAV-FLEX-GFP (green) and the dorsal dentate gyrus with a retrograde tracer (Fast Blue, blue). (F) Proportion of neurons expressing the reporter gene (GFP) that were back labeled by the injection of retrograde tracer into the dentate gyrus (74.3% ± 4.4%; 331/441 cells; 8 sections). Grey dots indicate percentage values calculated for each tissue section. Error bar represents SEM.

Sim1:Cre mice have been used previously to target reelin-positive stellate cells in L2 of the medial entorhinal cortex (MEC) [19, 20]. We therefore investigated whether Sim1:Cre mice also provide specific access to the reelin-positive fan cells in layer 2 (L2) of the lateral entorhinal cortex (LEC) (Figure S1). We found that, following injection of Cre-dependent adeno-associated virus (AAV)-encoding GFP (AAV-FLEX-GFP) [21] into the superficial LEC of Sim1:Cre mice (n = 4), the majority of cells expressing GFP were located in L2a of the LEC (Figures 1A and 1B). Staining with antibodies against reelin and calbindin revealed that expression of GFP was specific to cells that were positive for reelin, but not calbindin (Figures 1C and 1D). A small number of cells were triple labeled by reelin, calbindin, and GFP (Figure S2). To establish whether the GFP-expressing population of neurons overlapped with the population of cells that project to the hippocampus, we injected a further cohort of Sim1:Cre mice (n = 3) with AAV-FLEX-GFP into superficial LEC and a retrograde tracer (Fast Blue) into the dorsal dentate gyrus (DG). The majority of neurons expressing GFP co-localized with neurons labeled by the retrograde tracer (Figures 1E and 1F). Therefore, Sim1:Cre mice provide genetic access to a population of neurons in LEC L2 that are positive for reelin and project to the hippocampus.

**Figure 2:**
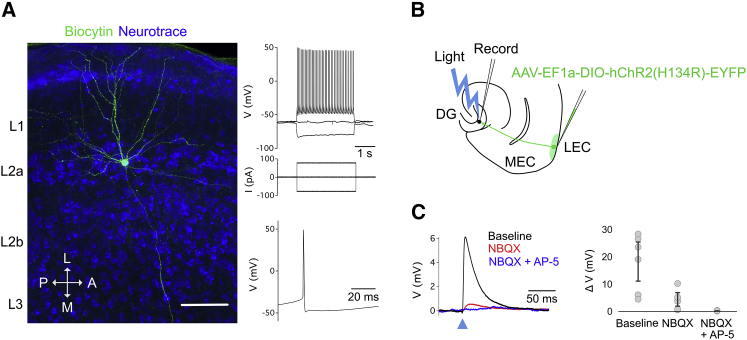
Neurons Labeled by Sim1:Cre Mice in Layer 2 of LEC Are Fan Cells (A) Representative example of the morphology and electrophysiology of a fan cell that expressed mCherry after injection of AAV-hSyn-DIO-hM4D(Gi)-mCherry into the superficial LEC of a Sim1:Cre mouse. See also Table S1. Left: the dendrites and axons of the cell are filled with biocytin (green), and neurons are counterstained with Neurotrace (blue). Note the axon leading toward the dentate gyrus. Scale bar represents 100 μm. Right: membrane potential response to the injection of negative and positive current steps (top) and example action potential (bottom) is shown. (B) Schematic of recording experiment to confirm that fan cells labeled in Sim1:Cre mice project to the dentate gyrus. AAV-EF1a-DIO-ChR2(H134R)-EYFP was injected into the superficial LEC of Sim1:Cre mice (n = 5). Synaptic output from fan cells in layer 2 of LEC was evaluated by recording light-evoked responses of granule cells in the dentate gyrus. (C) Membrane potential response of a dentate gyrus granule cell after optogenetic activation of fan cells in layer 2 of LEC expressing ChR2. Left: the peak response was abolished by application of ionotropic glutamate receptor antagonists NBQX (red) and AP-5 (blue). Right: quantification of the light-evoked membrane potential response of dentate gyrus granule cells (n = 6 cells, 5 mice) after application of NBQX and AP-5 is shown. Each gray circle represents an individual neuron. Error bars are SEM.

L2 of LEC contains several morphologically and electrophysiologically distinct subtypes of neurons, including pyramidal, multi-form, and fan cells [18, 22, 23]. To establish whether the population of cells labeled in Sim1:Cre mice mapped onto a specific subtype, we injected AAV encoding a Cre-dependent fluorescent reporter (AAV-hSyn-DIO-hM4D(Gi)-mCherry) into the superficial LEC of Sim1:Cre mice (n = 5) and performed patch-clamp recordings in brain slices from labeled cells in L2a of LEC (n = 15 cells). The electrophysiological properties of the labeled cells were consistent with those previously described for fan cells (Figure 2A; Table S1), including a relatively depolarized resting membrane potential (−68.5 ± 1.4 mV), high input resistance (130.5 ± 11.9 mΩ), slow time constant (24.2 ± 1.7 ms), and a “sag” membrane potential response to injection of hyperpolarizing current (0.85 ± 0.1) [18, 22]. Reconstruction of a subset of these cells (n = 12) revealed that their dendritic architecture was similar to previous descriptions of fan cells, with a polygonal soma and “fan-like” arrangement of primary dendrites. To test whether these cells provide synaptic output to the hippocampus, we injected AAV-encoding Cre-dependent channelrhodopsin-2 (ChR2; AAV-EF1a-DIO-hChR2(H134R)-EYFP) into the superficial LEC of Sim1:Cre mice (n = 5) to enable their optical activation (Figure 2B). We then recorded light-evoked glutamatergic synaptic potentials in downstream granule cells of the dentate gyrus in slices (Figure 2C; n = 6 cells), confirming that the labeled cells mediate perforant path inputs from the LEC to the dentate gyrus. Together, these experiments demonstrate that neurons expressing Cre in the LEC of Sim1:Cre mice are fan cells. Our analyses further confirm that fan cells express reelin and project to the dentate gyrus of the hippocampus.

### Fan Cells in L2 of LEC Are Critical for Episodic-like Memory

**Figure 3:**
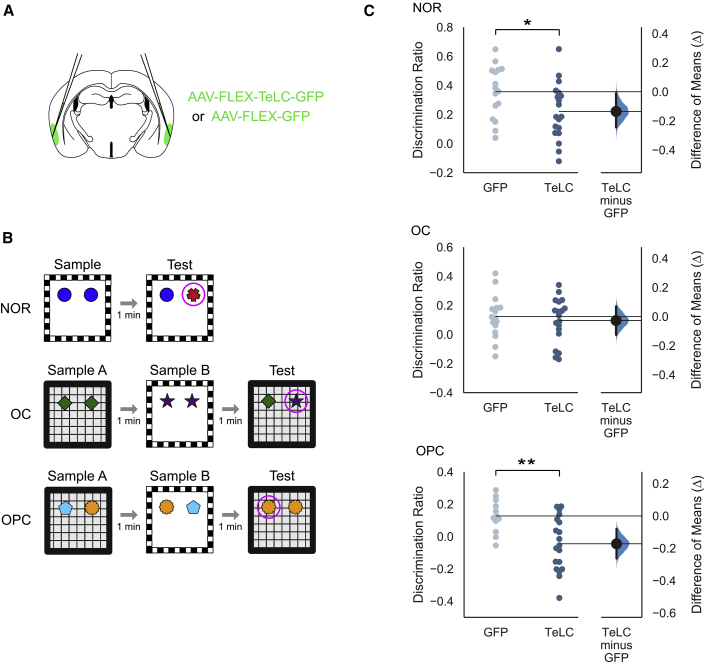
Suppression of Fan Cells in L2 of LEC Impairs Performance on the Object-Place-Context Task (A) Schematic of injection strategy used to bilaterally target superficial LEC with AAV-FLEX-TeLC-GFP or AAV-FLEX-GFP. (B) Schematic of object recognition tasks. Tasks included novel object recognition (NOR; top), object-context (OC; middle), and object-place context (OPC; bottom). Purple circles highlight the novel object or configuration in the test trial. (C) Discrimination ratios for each task. Discrimination ratios reflect the proportion of time spent exploring the novel object or configuration. Left: discrimination ratios for each group are plotted as swarm plots where each dot indicates the value for a single animal in the GFP control group (light blue) and TeLC group (dark blue). Right: Gardner-Altman estimation plots display effect size as the mean difference between the GFP control and TeLC groups (Δ). Δ is plotted as a black dot on a curve that indicates the resampled distribution of Δ, given the observed data. The 95% confidence interval of Δ is indicated by the ends of the vertical error bar. One-sample Wilcoxon signed rank tests against a median value of 0 revealed that mice in both groups performed significantly above chance on the NOR task (top, GFP control: *Z* = −4.170, p < 0.001; TeLC: *Z* = −3.834, p < 0.001) and the OC task (middle, GFP control: *Z* = −2.864, p = 0.004; TeLC: *Z* = −2.410, p = 0.016). In contrast, mice in the TeLC group showed no exploratory preference for the novel configuration in the OPC task (*Z* = −1.051; p = 0.294), whereas mice in the control group explored the novel configuration more than predicted by chance (*Z* = −2.927; p = 0.003). Comparisons with Mann-Whitney *U* revealed a significant difference in performance between TeLC mice and GFP control mice for the NOR (p = 0.045) and OPC tasks (p = 0.006), but not the OC task (p = 0.753). Asterisks indicate a significant difference between groups (^∗^p < 0.05; ^∗∗^p < 0.01). See also Figure S3.

Complete lesions of the LEC impair the ability to associate the different features of an episode [2, 3]. To test whether fan cells in L2 of LEC are required to form these associations, we blocked their output using targeted injections of AAV encoding a Cre-dependent tetanus toxin light chain (TeLC) into L2 of the LEC (Figure 3A; AAV-FLEX-TeLC-GFP), an approach we previously validated for inactivation of stellate cells in L2 of the MEC [20]. We compared the performance of Sim1:Cre mice injected with AAV-FLEX-TeLC-GFP (n = 21) with corresponding control mice injected with AAV-FLEX-GFP (n = 17) on a series of object-based memory tasks (Figure 3B) that model the integration of episodic memory components in rodents [24]. In the sample phase of each task, animals encountered two objects in different spatial locations within an environmental context that has distinct visual and tactile features. After a short interval, the animal was presented with a familiar object and an object that was novel or in a novel configuration of location and/or context.

Mice expressing TeLC in fan cells were able to distinguish novel objects and object-context configurations. Both TeLC and control mice explored novel objects more than expected by chance (TeLC: *Z* = −3.834, p < 0.001; GFP: *Z* = −4.170, p < 0.001), although discrimination ratios were lower for the TeLC group (Figure 3C; Mann-Whitney *U* = 213; p = 0.045). Novel object-context configurations were also explored more than expected by chance by the TeLC and control mice (TeLC: *Z* = −2.410, p = 0.016; GFP: *Z* = −2.864, p = 0.004) with no difference between the two groups (Mann-Whitney *U* = 162; p = 0.753). These data suggest that, although fan cells are not required for novel object recognition, outputs from fan cells may contribute to novel object discrimination. However, unlike the LEC as a whole, fan cells are not required for recognition of novel object-context associations.

When we investigated discrimination of more complex associations of object, place, and context, we found that mice in the TeLC group showed no exploratory preference for the novel configuration (*Z* = −1.051; p = 0.294), whereas mice in the control group explored the novel configuration more than predicted by chance (*Z* = −2.927; p = 0.003). Consistent with this, there was a significant difference in performance between the two groups (Mann-Whitney *U* = 183; p = 0.006). The amount of time spent exploring the objects was similar across groups for all tasks, indicating that differences between groups are not explained by reduced interest in the objects (Figure S3).

**Figure 4:**
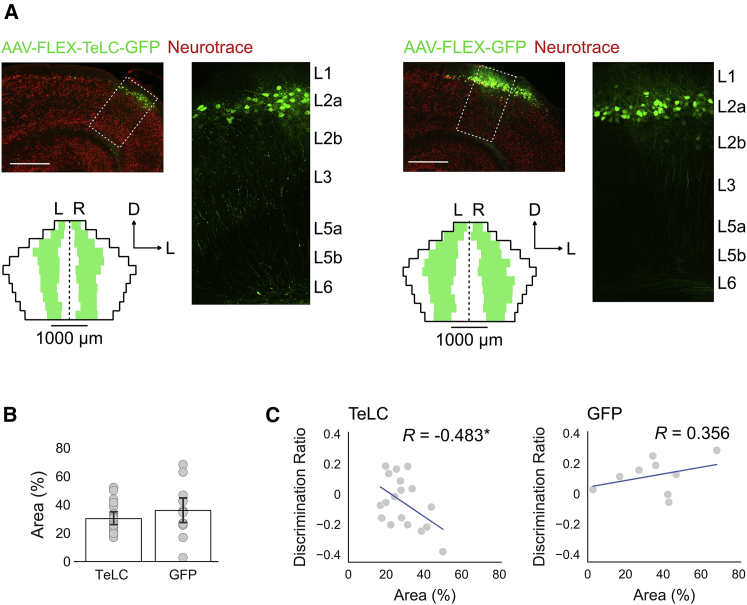
Quantification of Virus Expression in Mice Injected with AAV-FLEX-TeLC-GFP or AAV-FLEX-GFP (A) Horizontal brain sections showing expression of AAV-FLEX-TELC-GFP (left) or AAV-FLEX-GFP (right) in a Sim1:Cre mouse (top left). Reporter gene expression (GFP, green) is shown against neurons counterstained with Neurotrace (red). Scale bars represent 250 μm. The dashed boxes indicate the regions of LEC that are shown in the insets (right). Unfolded representations of LEC L2a (bottom) are overlaid with the average location and spread of virus expression for each group. Average distances between regions of virus expression and the borders of LEC (white) and average lengths of virus expression (green) were calculated by averaging measurements across all animals for sections at each dorsoventral coordinate of LEC (D, dorsal; L, lateral). Left and right hemispheres are indicated by L and R, and black dotted line indicates separation between hemispheres. (B) Bars indicate average percentage area of L2 of LEC that contained neurons that expressed the reporter gene after injection of AAV-FLEX-TELC-GFP or AAV-FLEX-GFP. Each gray dot represents the data for a single animal. Error bars are SEM. (C) Relationship between virus expression and performance for the OPC task. Scatterplots of the percentage area of virus expression in LEC L2 plotted against the discrimination ratios for the OPC task are shown. Each gray dot represents data for a single animal. Pearson’s correlation coefficients (*R*) were calculated to determine the strength of the relationship between discrimination ratios and virus expression. There was a significant negative correlation between discrimination ratios and virus expression in the TeLC group (p = 0.042), but not the GFP group (p = 0.347). Asterisks indicate a significant correlation between discrimination ratios and virus expression (^∗^p < 0.05). Each plot is overlaid with the line of best fit (blue) that was calculated using the least-squares method of linear regression. See also Figure S4.

If deficits in object-place-context recognition result from expression of TeLC, then performance should correlate with the extent to which AAV-FLEX-TeLC-GFP infected fan cells across the LEC. To test this, we quantified for each animal the proportion of L2 of LEC in which virus was expressed (Figures 4A and 4B). The pattern of GFP labeling in the older mice used for behavioral experiments was similar to that of younger mice used for electrophysiological and anatomical experiments (cf. Figures 1A and 4A). When we compared the percentage area of virus expression with the discrimination ratios calculated for object-place-context recognition, we found a significant negative correlation for the TeLC group (Figure 4C; *R* = −0.483; n = 18; p = 0.042), but not for the GFP group (*R* = 0.356; n = 9; p = 0.347). There was no significant correlation between virus expression and performance for the other recognition tasks (Figure S4).

## Discussion

Our data provide evidence that fan cells in L2 of LEC are critical for episodic-like memory of object-place-context associations but are not required to integrate information about objects and context. These findings are in contrast to general associative memory impairments caused by complete lesions of the LEC [2, 3]. Our results suggest that complex information about objects in the environment encoded by neurons in the LEC [7, 8, 9, 10, 11] is used in multiple ways. Output from fan cells mediates formation of object-place-context associations, whereas other LEC cell types contribute to simpler object-context associations.

This functional specialization within the LEC has implications for normal circuit computations and disorders. Our results suggest a model in which L2a of LEC generates outputs that are specifically required for relatively complex object-place-context associations, whereas neurons in other layers may be sufficient for formation of simpler associations. Impaired episodic-like memory but spared recognition of simpler associations following lesions of the hippocampus, which receives input from fan cells, is consistent with this possibility [1]. Thus, object-place-context associations may involve hippocampal processing of fan cell outputs before affecting behavior. Cell populations in the LEC that are upstream of fan cells may integrate object and context information and could influence object-context behaviors independently from the hippocampus through output neurons in deep layers of LEC [25].

Our results are consistent with the idea that hippocampal circuitry immediately downstream of the LEC also has dissociable memory functions. Numerous studies have demonstrated a role for DG in pattern separation [26, 27, 28, 29, 30], and CA1 has been implicated in processing temporal information [30, 31, 32, 33, 34], orthogonalization of spatial inputs [35, 36], and encoding contextual and object cues within the environment [37, 38, 39, 40, 41, 42, 43]. Synaptic inputs from fan cells to DG may be important for complex discriminations between objects with overlapping contextual and spatial features, consistent with the deficits that we describe here. In contrast, representations of time and objects generated by LEC neurons [7, 8, 9, 10, 11, 12] may reach CA1 through direct input from layer 3 of the LEC as well as indirect input via the DG and CA3. These inputs could contribute to a network that encodes temporal and contextual features of an episode, consistent with intact object-context recognition after inactivation of fan cells.

An alternative interpretation of our data, suggested by the significant difference in novel object recognition between control mice and mice expressing TeLC in fan cells, is that fan cells play a more general role in novel object recognition. However, several observations indicate that this explanation is unlikely. First, the TeLC-expressing group showed a clear preference for novel objects, indicating that fan cells are not required for novel object recognition. Second, the TeLC group performed similarly to the control group in the object-context recognition task, demonstrating that the small difference between the groups in novel object recognition does not influence object-context associations and therefore is also unlikely to affect associations between objects, context, and place. Third, lesion studies indicate that the LEC as a whole is not required for novel object recognition, although considerable evidence suggests that the upstream perirhinal cortex mediates recognition of novel objects [44]. The deficits we find in object-place-context associations made by the TeLC-expressing group are therefore unlikely to result from a deficit in novel object recognition.

The dissociation we find between roles of fan cells in object-place-context and object-context associations raises questions about the assembly of multi-modal associations within the LEC. For example, deficits in object-place-context association could result from a failure to distinguish the two identical objects based on either their location or context or they could result from a failure to fully integrate object, place, and context information. Whether object-place associations can be determined independently from contextual changes and without engagement of fan cells remains to be determined. A critical issue will be to identify the synaptic pathways through which place signals are integrated with object and contextual information within the LEC.

An impaired ability to associate different features in memory is characteristic of early Alzheimer’s disease (AD) [45, 46, 47, 48], and our findings are consistent with correlations between pathology of the superficial entorhinal cortex and early cognitive deficits in AD and in age-related cognitive decline [13, 15, 16, 49, 50]. By showing that episodic-like memory requires output from fan cells, our results suggest that pathology specific to L2a of LEC can result in associative memory impairments reminiscent of those observed in the early stages of AD.

## STAR★Methods

### Key Resources Table

**Table:** 

REAGENT or RESOURCE	SOURCE	IDENTIFIER
**Antibodies**		

Mouse anti-reelin	MBL	Cat # D351-3; RRID: AB_2815002
Rabbit anti-calbindin D-28K	SWANT	Cat # CB-38; RRID: AB_10000340
AlexaFluor Goat Anti-Mouse 488	Invitrogen	Cat # A-10680; RRID: AB_2534062
AlexaFluor Goat Anti-Rabbit 555	Invitrogen	Cat # A-11003; RRID: AB_141370
AlexaFluor Goat Anti-Rabbit 647	Invitrogen	Cat # A27040; RRID: AB_2536101
Neurotrace 640/660	Invitrogen	Cat # N21483; RRID: AB_2572212
AlexaFluor Streptavidin 488	Invitrogen	Cat # S32354; RRID: AB_2315383

**Bacterial and Virus Strains**		

AAV2-hSyn-DIO-hM4D(Gi)-mCherry	UNC Vector Core	Lot No: AV44362
AAV2-EF1a-DIO-hChR2(H134)-EYFP	UNC Vector Core	Lot No: AV43780
AAV-Flex-GFP	[21]	NA
AAV-Flex-GFP-TeLC	[21]	NA

**Chemicals, Peptides, and Recombinant Proteins**		

Fast Blue	Polysciences	17740
Biocytin	Sigma Aldrich	B4261
NBQX disodium salt	Hello Bio	HB0443
D-AP5	Hello Bio	HB0225

**Experimental Models: Organisms/Strains**		

Tg(Sim1-cre)KJ21Gsat/mmcd	GENSAT/ MMRRC	https://www.mmrrc.org/catalog/sds.php?mmrrc_id=34614

**Software and Algorithms**		

MATLAB	Mathworks	https://www.mathworks.com/products/matlab
Igor Pro	Wavemetrics	https://www.wavemetrics.com/
AxoGraph	AxoGraph	https://axograph.com/
NeuroMatic	[51]	http://www.neuromatic.thinkrandom.com/
ZenPro	Zeiss	https://www.zeiss.com/microscopy/int/products/microscope-software/zen.html
NIS Elements	Nikon	https://www.microscope.healthcare.nikon.com/en_EU/products/software/nis-elements
ImageJ	Fiji	https://fiji.sc/

**Other**		

Dual Lock Fastener	3M	SJ3560

### Lead Contact and Materials Availability

Further information and requests for resources and reagents should be directed to, and will be fulfilled by the Lead Contact, James Ainge (jaa7@st-andrews.ac.uk). This study did not generate new unique reagents.

### Experiment Model and Subject Details

#### Animals

The Sim1:Cre line, which expresses Cre under the control of the Single minded homolog-1 (Sim1) promoter, was generated by GenSat and obtained from MMRRC (strain name: Tg(Sim1cre)KH21Gsat/Mmucd). Sim1:Cre mice were bred to be heterozygous for the Cre transgene by crossing a male Sim1:Cre mouse carrying the transgene with female C57BL6/J mice. All mice were housed in groups in diurnal light conditions (12-hr light/dark cycle) and had *ad libitum* access to food and water. Anatomical and electrophysiological experiments used 5-11 week old C57BL6/J (n = 4, 2 male) and Sim1:Cre mice (n = 14, 5 male). Behavioral experiments used 3-8 month old Sim1:Cre mice (n = 38; TeLC = 21, 10 male; GFP = 17, 8 male). The age of the mice was comparable between groups (TeLC: 4.99 ± 0.67 months, GFP control: 4.38 ± 0.88 months, *t*(36) *=* 0.573, p = 0.570). To control for order and cage effects, each cage contained a mixture of mice from the experimental and control group. All experiments and surgery were conducted under project licenses administered by the UK Home Office and in accordance with national (Animal [Scientific Procedures] Act, 1986) and international (European Communities Council Directive of 24 November 1986 (86/609/EEC) legislation governing the maintenance of laboratory animals and their use in scientific research.

### Method Details

#### Stereotaxic Injection of Tracers and Viruses

Mice were anaesthetised with Isoflurane in an induction chamber before being transferred to a stereotaxic frame. Mice were administered an analgesic subcutaneously, and an incision was made to expose the skull. For retrograde labeling of neurons which project to the hippocampus, Fast Blue (Polysciences, Hirschberg an der Bergstraße, Germany) was injected unilaterally into the dorsal dentate gyrus. A craniotomy was made at 2.8 mm posterior to bregma and 1.8 mm lateral to the midline. A glass pipette was lowered vertically into the brain to a depth 1.7 mm from the surface, and 50-100 nL of tracer diluted at 0.5% w/v in dH_2_O was injected. We used the following adeno-associated viruses for injections into lateral entorhinal cortex: AAV-1/2-FLEX-GFP and AAV-1/2-FLEX-TeLC-GFP (generated in house following protocols described by Murray et al., 2011 [21]), AAV2-hSyn-DIO-hM4D(Gi)-mCherry and AAV2-EF1a-DIO-hChR2(H134R)-EYFP (UNC Vector Core, Chapel Hill, North Carolina). To target the lateral entorhinal cortex, a craniotomy was made adjacent to the intersection of the lamboid suture and the ridge of the parietal bone, which was approximately 3.8 mm posterior to bregma and 4.0 mm lateral to the midline. From these coordinates, the craniotomy was extended 0.8 mm rostrally. At the original coordinates, a glass pipette was lowered from the surface of the brain at a 10°-12°angle until a slight bend in the pipette indicated contact with the dura. The pipette was retracted 0.2 mm and 150-500 nL of virus was injected. This protocol was repeated at a site 0.2 mm rostral to this site. To target ventral lateral entorhinal cortex, the angle of the pipette was adjusted to 6° - 9° and a third injection was delivered at the rostral injection site. The angle was adjusted within each reported range based on the proximity of the craniotomy to the ridge of the parietal bone. This approach minimized the likelihood of spread of virus into adjacent cortical structures. For all injections, the pipette was slowly retracted after a stationary period of four minutes. Mice were administered an oral analgesic prepared in flavoured jelly after surgery. For behavior experiments, mice were habituated to the test environment one week after surgery, and testing commenced two weeks after surgery.

#### Immunohistochemistry

For tracer injections, animals were sacrificed 1-2 weeks post-surgery. For anatomical experiments animals were sacrificed 2-4 weeks post-surgery. Mice were administered a lethal dose of sodium pentobarbital and transcardially perfused with cold phosphate buffered saline (PBS) followed by cold paraformaldehyde (PFA, 4%). Brains were extracted and fixed for a minimum of 24 hours in PFA at 4°C, washed with PBS, and transferred to a 30% sucrose solution prepared in PBS for 48 hours at 4°C. Brains were sectioned horizontally at 60 μm on a freezing microtome. For staining against reelin and calbindin, slices were blocked for 2-3 hours in 5% Normal Goat Serum (NGS) prepared in 0.3% PBS-T (Triton). Slices were then transferred to a primary antibody solution prepared in 1% NGS in 0.3% PBS-T for 24 hours. Primary antibodies were mouse anti-reelin (MBL, 1:200) and rabbit anti-calbindin D-28K (SWANT, 1:2500). Slices were washed with 0.3% PBS-T 3x for 20 minutes before being transferred to a secondary antibody solution prepared in 0.3% PBS-T for 24 hours. Secondary antibodies were AlexaFluor® Goat Anti-Mouse 488 and 546 and AlexaFluor® Goat Anti-Rabbit 555 and 647 (Invitrogen, all used at 1:500). Neurotrace 640/660 (Invitrogen, 1:800) was included in the secondary antibody solution as a counterstain. Slices were washed with 0.3% PBS-T 3x for 20 minutes and then mounted and coverslipped with Mowiol. Mounted sections were stored at 4°C.

#### Slice Electrophysiology

Horizontal brain slices were prepared from 5-11 week old Sim1:Cre mice 2-4 weeks after injection of AAV-hSyn-DIO-hM4D(Gi)-mCherry or AAV-EF1a-DIO-hChR2(H134R)-EYFP into superficial LEC. Mice were sacrificed by cervical dislocation and decapitated. The brains were rapidly removed and submerged in cold cutting artificial cerebrospinal fluid (ACSF) at 4-8°C. The cutting ACSF was comprised of the following (in mM): NaCl 86, NaH_2_PO4 1.2, KCl 2.5, NaHCO_3_25, Glucose 25, Sucrose 50, CACl_2_ 0.5, MgCI_2_ 7. The dorsal surface of the brain was glued to a block submerged in cold cutting ACSF and horizontal slices were cut using a vibratome at a thickness of 300-400 μm. Slices were transferred to standard ACSF at 37°C for 15 minutes, then incubated at room temperature for a minimum of 45 minutes. Standard ACSF was comprised of the following (in mM): NaCl 124, NaH_2_PO4 1.2, KCI 2.5, NaHCO_3_ 25, Glucose 25, CaCI_2_ 2, MgCI_2_ 1. For recordings, slices were transferred to a submerged chamber and maintained in standard ACSF at 35-37°C. Labeled neurons in LEC were identified by their expression of the relevant fluorophore. To target cells in the dentate gyrus which receive input from LEC, granule cells were patched in regions of the granule cell layer where there was virus expression in axon fibers from LEC in the corresponding region of the outer molecular layer. Whole cell patch-clamp recordings were made using borosilicate electrodes with a resistance of 3-6 MΩ for cells in LEC and 6-10 MΩ for cells in dentate gyrus. Electrodes were filled with an intracellular solution comprised of the following (in mM): K gluconate 130, KCI 10, HEPES 10, MgCl_2_2, EGTA 0.1, Na_2_ATP 4, Na_2_GTP 0.3, phosphocreatine 10, and 0.5% biocytin (w/v). Recordings were made in current-clamp mode from cells with series resistance ≤ 50 MΩ with appropriate bridge-balance and pipette capacitance neutralisations applied.

#### Recording Protocols

A series of protocols were used to characterize the electrophysiological properties of each cell recorded in LEC or the dentate gyrus, as described previously [52, 53]. Sub-threshold membrane properties were measured by examining membrane potential responses to injection of current in hyperpolarizing and depolarizing steps (−160 to 160 pA in 80 pA increments, or −80 to 80 pA in 40 pA increments, each 3 s duration), and to injection of an oscillatory current with a linearly varying frequency (ZAP protocol). Supra-threshold properties were estimated from responses to depolarizing current ramp applied to the cell in a constantly increasing manner to induce action potentials (50 pA/s, 3 s). Responses to optogenetic activation of LEC fiber inputs to dentate granule cells were evaluated by stimulation with 470 nm wavelength light for 3 ms at 22.4 mW/mm^2^. After recording a baseline response to light stimulation, ionotropic glutamate receptor antagonists for AMPA (NBQX, 10 μM) and NMDA receptors (AP-5, 50 μM) were bath applied, and the response to light stimulation was re-evaluated using the same stimulation protocols. Upon completion of investigatory protocols, diffusion of biocytin into the cell was encouraged by injecting large depolarizing currents into the cell (15 × 4 nA, 100 ms steps, 1Hz) [54]. Each cell was left stationary with the electrode attached for a maximum of one hour before being transferred to PFA (4%) and stored at 4°C for at least 24 hours before histological processing.

#### Neuron Reconstruction

To reveal the morphology of biocytin filled neurons, fixed slices were washed with PBS 4 times for 10 minutes and transferred to a solution containing AlexaFluor® Streptavidin 488 (Invitrogen, 1:1000 or 1:500) and Neurotrace 640/660 (Invitrogen, 1:1000 or 1:500) in 0.3% PBS-T for 24 hours. Slices were washed with PBS-T 4 times for 20 mins and then mounted and coverslipped with Mowiol. Mounted sections were stored at 4°C.

#### Behavioral Apparatus

The test environment was a rectangular wooden box (length 32 cm, width 25.5 cm, height 22 cm) that could be configured to provide two distinct contexts. Context A had black and white vertically striped walls with a smooth white floor. Context B had gray walls with a wire-mesh floor. The environment was lit by two lamps positioned equidistantly from the box. The wall and floor of the environment was cleaned with veterinary disinfectant before each trial. To secure objects in place within the environment, square sections of fastening tape (Dual Lock, 3M) were positioned in each of the upper left and right quadrants of the box floor. Objects were household items of approximately the same size as a mouse and varying in color, shape, and texture. Objects were matched for similarity in size and complexity when paired for testing. To avoid the use of odour cues, new identical copies of each object were used for each trial, and objects were cleaned with disinfectant after each trial.

#### Design of Behavioral Experiments

Behavioral experiments were carried out in two cohorts of mice. In the main manuscript data from the cohorts are pooled. The experimenter was blind to the manipulation (delivery of either AAV-FLEX-TeLC-GFP or AAV-FLEX-GFP) during all behavioral experiments and their initial analyses. Tetanus toxin light chain (TeLC) was chosen for our manipulations instead of a within-subject approach such as inhibitory DREADDs (Designer Receptors Exclusively Activated by Designer Drugs) due to concerns that the stress induced by intraperitoneal delivery of the associated ligand has adverse effects on spontaneous object recognition behavior.

#### Habituation

In the week before surgery, the experimenter handled each animal every day for 10 minutes in the testing room. Habituation to the test environment commenced one week after surgery and lasted for 5 consecutive days. On day 1, the mice explored each context for 10 minutes with their cage mates. On days 2-5 the mice explored each context for 10 minutes individually. On days 4-5, objects were introduced in the upper left and right quadrants of the test environment. Objects used during habituation were not re-used during testing.

#### Behavioral Tasks

Testing commenced in the week after habituation and occurred in three stages: Novel object recognition (NOR), novel object-context (OC) recognition, and novel object-place-context (OPC) recognition. Novel object-place recognition was also included as a stage in these experiments, but neither experimental group discriminated the novel object-place configuration at a level above chance, therefore we excluded it from further analysis. Each stage lasted for 4 days. For all sample and test trials, the animal was allowed to explore the environment freely for 3 mins. Between trials, mice were removed to a holding cage for approximately 1 minute while the test environment was configured for the subsequent trial. For each task, the object that was novel at test, the context, and the quadrants where the novel object or configuration occurred were counterbalanced across animals and experimental conditions. For OC and OPC tasks, the presentation order of context A and B in the sample phase, the context used at test, and the context in which each object was presented during the sample phase were also counterbalanced across animals and conditions. The stages are described here in the order in which they occurred:

1 NOR Task: In the sample trial, mice were presented with two copies of a novel object in one of the contexts (striped or gray walls). In the test trial, mice were presented with a new copy of the object from the sample trial (now familiar) and a novel object in the same context as the sample trial.
2 OC task: In the first sample trial, mice were presented with two copies of a novel object in context A. In a second sample trial, mice were presented with two copies of a different novel object in context B. In the test trial, mice were presented with a single copy of each object within one of the contexts (A or B). At test, both objects are familiar and have been encountered at both locations, but one object has previously been encountered in the test context (familiar OC configuration), whereas the other one has not been encountered in the test context (novel OC configuration).
3 OPC task: In the first sample trial, mice were presented with two different novel objects in context A. In a second sample trial, mice were presented with the same objects in opposite locations in context B. In the test trial, mice were presented with two copies of one of the objects within one of the contexts (A or B). At test, one copy of the object is in a novel location for the test context (novel OPC configuration), whereas the other copy is in a familiar location for the test context (familiar OPC configuration).

#### Histology

For behavioral experiments, mice were sacrificed 6-8 weeks post-surgery. Mice were transcardially perfused as described previously. Slices were washed in 0.3% PBS-T 3 times for 20 minutes before being transferred to a solution containing Neurotrace 640/660 (Invitrogen, 1:800) prepared in 0.3% PBS-T for 2-3 hours at room temperature. Slices were washed with 0.3% PBS-T 3 times for 20 minutes before being mounted and coverslipped with Mowiol. Mounted sections were stored at 4°C.

#### Microscopy

For tracer and anatomical experiments, images were acquired using a Nikon A1 confocal microscope and NIS elements software. For quantification of cells immunolabelled for reelin or calbindin, or labeled by retrograde tracer or fluorescent reporter, 20x z stacks were acquired of regions of interest (ROI) at 1–2 μm steps. ROIs were regions of L2 of various sizes within the borders of LEC, which were determined by referencing an atlas of the mouse brain [55]. For reconstruction of neurons after recording in slices, z stacks were acquired of filled cells at 1-2 μm steps using a 20x objective on a Nikon A1 confocal microscope and NIS elements software or a Zeiss LSM800 confocal microscope and ZenPro software. The morphology of cells recorded in LEC were confirmed by visual comparison of the shape of the soma and arrangement of dendrites to published morphological descriptions [18, 22, 23]. For quantification of the location and extent of virus expression after behavior experiments, images were acquired of 1:4 serial sections at 10x magnification using a fluorescent microscope (Zeiss ApoTome) and ZenPro software.

### Quantification and Statistical Analysis

#### Quantification of Immunohistochemistry

For quantification of labeling by an antibody, tracer or fluorescent reporter, all neurons within each imaged ROI were counted manually. Fractions of labeled cells were determined by calculating for each ROI the number of labeled cells divided by the total number of Neurotrace labeled cells. Proportions were averaged across all ROIs from all mice to yield an overall percentage of labeled cells (Figures 1B, 1D, and 1F).

#### Analysis of Electrophysiological Data

Electrophysiological data were analyzed with AxoGraph (https://axographx.com), IGOR Pro (Wavemetrics, USA) using Neuromatic (http://www.neuromatic.thinkrandom.com) [51], and customised MATLAB scripts. Input resistance, time constant and time-dependent inward rectification (‘sag’) were measured from the membrane potential response to injections of hyperpolarizing current (−80 or −40 pA). Input resistance (MΩ) was estimated by dividing the steady-state voltage change from the resting membrane potential by the amplitude of the injected current, time constant (ms) was measured as the time taken for the change in voltage to reach a level 37% above its maximal decrease, and ‘sag’ was measured as the ratio between the maximum decrease in voltage and the steady-state decrease in voltage. Rheobase (pA) was measured as the minimum amplitude of depolarizing current which elicited an action potential response from the cell. Action potential duration (ms) was measured from the action potential threshold (mV), which was defined as the point at which the first derivative of the membrane potential voltage that exceeded 1 mV/1 ms. Action potential amplitude (mV) was measured as the change in voltage between the action potential threshold and peak. To determine the resonant frequency of the cell, membrane impedance was first calculated by dividing the Fourier transform of the membrane voltage response by the Fourier transform of the input current from the ZAP protocol, which was then converted into magnitude and phase components. The resonant frequency was defined as the input frequency which corresponded to the peak impedance magnitude. The results of these analyses can be found in Table S1. Values are presented as population means. To quantify the response of granule cells to optical stimulation of fiber input from LEC, the change in amplitude was measured between the resting membrane potential and peak response during stimulation (Figure 2C).

#### Quantification of Virus Expression in Behavioral Animals

To confirm the location and extent of virus expression in each behavioral animal, coordinates for each section were determined by referencing an atlas of the mouse brain [55]. Unfolded representations of LEC L2a were generated by adapting procedures previously used to quantify lesions of the entorhinal cortex [56, 57]. The anterior and posterior borders of the LEC were determined by the bifurcation of L2, which is absent in adjacent cortical structures. A subset of brains (n = 1 TeLC, 4 GFP) suffered mechanical damage to LEC L2 in > 30% of sections that contained LEC. Removal of these mice from the dataset did not change the interpretation of statistical comparison between groups, therefore they were included in the analysis of behavior but excluded from analyses which address the relationship between virus expression and behavior. The length of L2a was measured in ImageJ (https://fiji.sc) using a built-in tool calibrated to the scale of the image. For sections with virus expression, three measurements were extracted: the distance of the region of expression from the anterior LEC border, the length of the region of expression, and the distance of the region of expression from the posterior LEC border. The region of expression was defined as the length of L2a between the most anterior infected neuron and the most posterior infected neuron. These measurements were used to calculate the proportion of LEC L2a in which the virus was expressed for each animal (Figure 4B) and averaged across all animals for sections with virus expression across the dorsoventral axis to obtain the mean values used to generate the unfolded representations shown in Figure 4A. Adjacent structures were examined for unintended expression of virus in the TeLC group. There was labeling in LEC L5a in a subset of mice (n = 11). In most cases (n = 8), this was negligible, summating to < 10 cells across all sections. In other cases (n = 3), this was < 5% of the area of LEC L5. Further, there was minor spread of virus to the region of MEC L2 directly adjacent to LEC in a subset of mice (n = 8), but this was < 5% of the area of MEC L2 in all cases. There was no significant difference between performance of mice with L5 expression or MEC expression and other mice in the TeLC group on any task.

#### Analysis of Behavioral Data

All trials were recorded by a camera positioned above the test environment. Footage was scored offline by an experimenter who was blind to experimental condition. To ensure reliability of the scores a random sample of 50% of the trials were rescored by a second experimenter who was also blind to condition. Reliability between scorers was good with an intraclass correlation coefficient of 0.878 (2-way mixed model). For each trial, the amount of time spent exploring each object was measured. Exploration was defined as periods where the animal’s nose is within 2 cm of the object and oriented toward the object, but the animal was not interacting with the object (e.g., biting) or rearing against the object to look out of the test environment. To determine whether the animal had an exploratory preference for the novel object or configuration, a discrimination ratio was calculated for each test trial [58]. The discrimination ratio is calculated by subtracting the amount of time spent exploring the object in the familiar configuration from the amount of time spent exploring the object in a novel configuration, and then dividing this value by the total exploration time. A positive discrimination ratio indicates an exploratory preference for the object in a novel configuration. For each animal, average discrimination ratios were calculated for each task. A population mean was then calculated for experimental (AAV-FLEX-TeLC-GFP) and control (AAV-FLEX-GFP) groups. Trials where the total exploration time was < 5 s during sample or test were excluded. Where ≥ 3 trials of a task met the criteria for exclusion for an animal, data from that animal was removed from the dataset for that task (NOR, n = 3, 1 TeLC and 2 GFP control; OC, n = 2 GFP control, OPC: n = 7, 2 TeLC and 5 GFP control).

#### Statistical Analysis of Behavioral Data

To determine whether the median discrimination ratios for each group were different from chance, one-sample Wilcoxon signed rank tests were conducted against a value of 0 for each task. To determine whether there was an effect of experimental group, Mann-Whitney *U* tests were conducted to compare discrimination ratios between groups for each task (see Figure 3C). Effect size was estimated by calculating the difference of means between groups (Δ) and deriving a 95% confidence interval by resampling the distribution 5000 times around Δ given the observed data. Effect size analyses were conducted using the open-source Data Analysis with Bootstrap-coupled ESTimation (DABEST) library for Python [59]. To determine whether there was a relationship between the extent of virus expression and behavior, Pearson’s product-moment correlation coefficients were calculated with percentage area of virus expression as a variable against the average discrimination ratio of each task for each animal (Figure 4C). Lines of best fit were calculated for the dataset using the least-squares method of linear regression.

### Data and Code Availability

Original data for the behavior experiments in the paper (Figures 3C, 4B, 4C, S3, and S4) is available at the University of Edinburgh Datashare Repository (https://doi.org/10.7488/ds/2629).

## Supplemental Information

Document S1. Figures S1–S4 and Table S1

Document S2. Article plus Supplemental Information
