# Supplementary material for: Fan Cells in Layer 2 of the Lateral Entorhinal Cortex Are Critical for Episodic-like Memory
Source: Curr Biol. 2020 Jan 6;30(1):169–175.e5. doi: 10.1016/j.cub.2019.11.027 (PMC6947484; doi:10.1016/j.cub.2019.11.027)
Supplement: Document S1. Figures S1–S4 and Table S1 [file mmc1.pdf]

Current Biology, Volume *30*

Supplemental Information

Fan Cells in Layer 2

# of the Lateral Entorhinal Cortex

Are Critical for Episodic-like Memory

Brianna Vandrey, Derek L.F. Garden, Veronika Ambrozova, Christina McClure, Matthew F. Nolan, and James A. Ainge

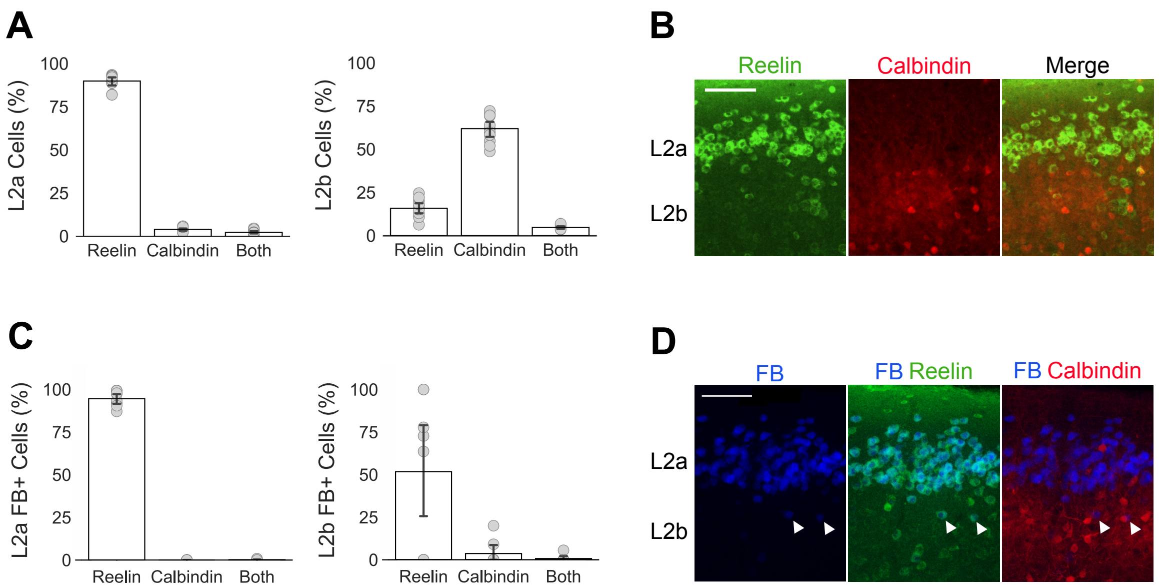

Figure S1. Quantification of reelin, calbindin and retrograde tracer labelling across sub-layers of LEC L2. Related to Figure 1.

A) Quantification of cells positive for reelin and calbindin in LEC L2a (left) and L2b (right; n = 4 mice). Note that the majority of cells in LEC L2a and L2b are positive for reelin and calbindin, respectively, as reported by Leitner et al. (2016) [S1]. Grey dots indicate percentage values calculated for each section of tissue. Error bars represent SEM. B) Immunolabelling against reelin (green) and calbindin (red) in L2a and L2b of LEC. C) Quantification of cells back-labelled by the retrograde tracer which were positive for reelin and calbindin in LEC L2a (left) and L2b (right; n = 4 mice). Note that the majority of back-labelled cells are positive for reelin in both sub-layers. Grey dots indicate percentage values calculated for each section of tissue. Error bars represent SEM. D) Immunolabelling against reelin (green) and calbindin (red) overlaid with neurons that were back-labelled by the injection of the retrograde tracer Fast Blue (FB) into the dentate gyrus (blue). White arrows indicate back-labelled neurons in LEC L2b which are positive for reelin. Scale bars represent 100 μm.

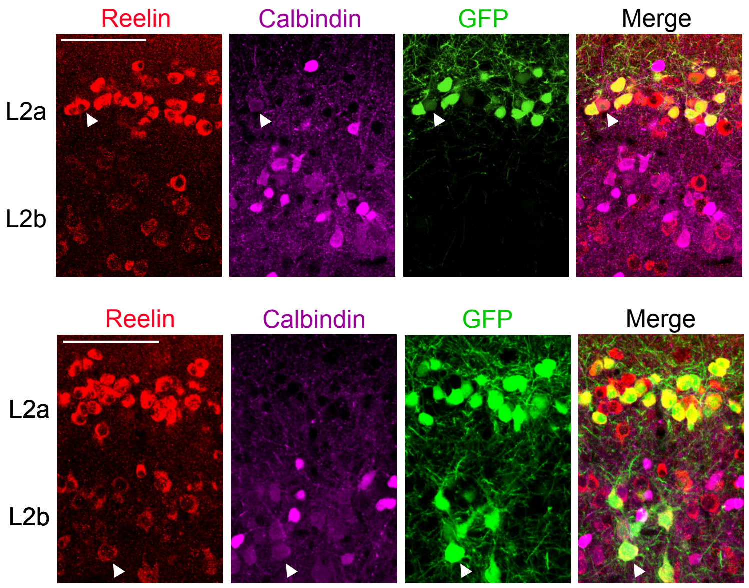

Figure S2. Triple-labelling of neurons in L2 of LEC. Related to Figure 1.

Cells triple-labelled by reelin (red), calbindin (purple) and the reporter gene (GFP, green) are indicated by white arrows. A small population of cells was triple-labelled in L2a (top, 0.3 ± 0.2%, 4/1282 cells) and L2b (bottom, 4.6 ± 1.9%, 13/207 cells). Scale bars represent 100 μm.

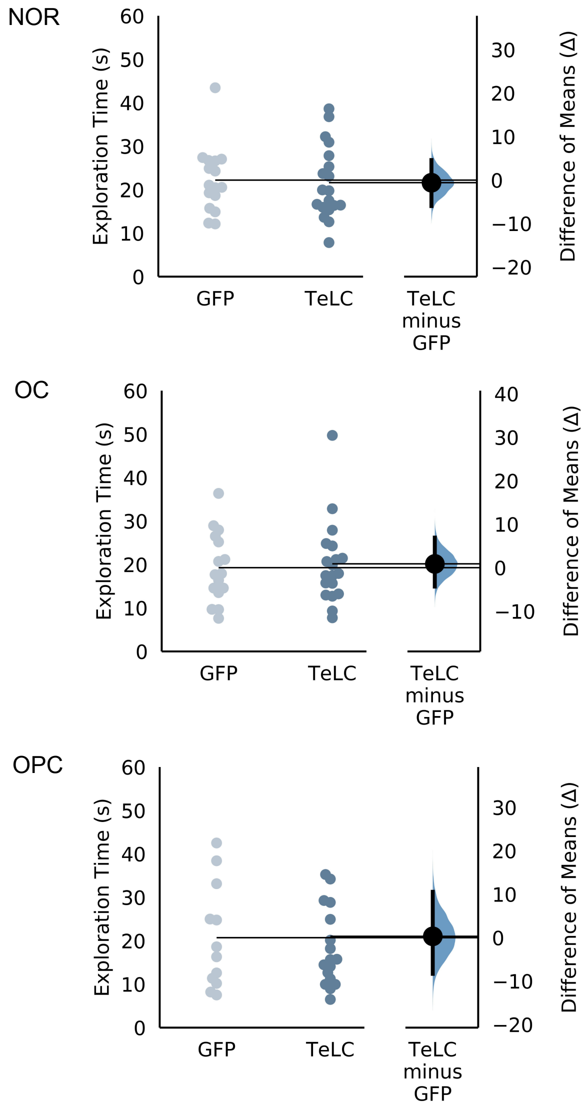

Figure S3. Exploration times for mice in the TeLC and GFP control group during the test trials of the novel object recognition (NOR), object-context (OC) and object-place-context (OPC) tasks. Related to Figure 3.

(Left) Values for each group are plotted as swarm plots where each dot indicates the time spent exploring the objects in seconds for a single animal in the GFP control group (light blue) and TeLC group (dark blue). (Right) Gardner-Altman estimation plots display effect size as the mean difference between the GFP control and TeLC groups (Δ). Δ is plotted as a black dot on a curve which indicates the resampled distribution of Δ, given the observed data. The 95% confidence interval of Δ is indicated by the ends of the vertical error bar.

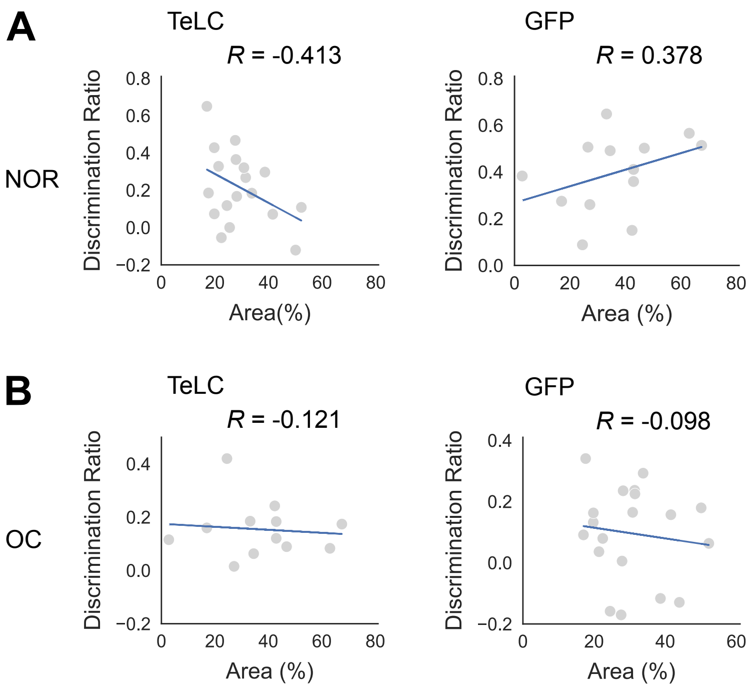

Figure S4. Relationship between virus expression and performance for novel object recognition (NOR) and object-context (OC) tasks. Related to Figure 4.

Scatterplots of the percentage area of virus expression in L2 plotted against the discrimination ratios for the NOR (A) and OC task (B). Each grey dot represents data for a single animal. Pearson's correlation coefficients (*R*) were calculated to determine the strength of the relationship between discrimination ratios and virus expression. The exact value of *R* is indicated in the top right corner of each plot. There was no significant correlation between discrimination ratios and virus expression in either group for the NOR (TeLC: $P = 0.089$; GFP control: $P = 0.202$) or OC task (TeLC: $P = 0.622$; GFP control: $P = 0.762$). Each plot is overlaid with line of best fit (blue) that was calculated using the least squares method of linear regression.

Property	Average	SD	SEM
Resting membrane potential (mV)	-68.47	5.22	1.35
Input resistance (mΩ)	130.47	45.97	11.87
Time constant (ms)	24.19	6.64	1.71
Sag	0.85	0.07	0.02
Rheobase (pA)	126.43	34.36	8.87
Actional potential threshold (mV)	-37.95	5.84	1.51
Action potential width (ms)	0.67	0.07	0.02
Action potential amplitude (mV)	88.19	3.65	0.94
Resonance frequency (Hz)	1.15	0.35	0.09
Resonance magnitude (Hz)	1.03	0.05	0.01

Table S1. Electrophysiological properties of fan cells. Related to Figure 2.

Table contains the population average, standard deviation (SD) and standard error of the mean (SEM) values for the electrophysiological properties of fan cells (n= 15 cells, 5 mice) in LEC L2 which expressed the reporter gene (mCherry) after injection of AAV-hSyn-DIO-hM4D(Gi)-mCherry into the superficial LEC.

Supplemental References

S1. Leitner, F.C., Melzer, S., Lütcke, H., Pinna, R., Seeburg, P.H., Helmchen, F., and Monyer, H. (2016). Spatially segregated feedforward and feedback neurons support differential odor processing in the lateral entorhinal cortex. Nature Neuroscience *19*, 935–944.
